# A Novel Bioinspired Algorithm for Mixed and Incomplete Breast Cancer Data Classification

**DOI:** 10.3390/ijerph20043240

**Published:** 2023-02-13

**Authors:** David González-Patiño, Yenny Villuendas-Rey, Magdalena Saldaña-Pérez, Amadeo-José Argüelles-Cruz

**Affiliations:** 1 Centro de Investigación en Computación, Instituto Politécnico Nacional, Ciudad de México 07738, Mexico; 2 Instituto Politécnico Nacional, Centro de Innovación y Desarrollo Tecnológico en Cómputo, Ciudad de México 07700, Mexico

**Keywords:** breast cancer, bio-inspired algorithms, machine learning, artificial intelligence

## Abstract

The pre-diagnosis of cancer has been approached from various perspectives, so it is imperative to continue improving classification algorithms to achieve early diagnosis of the disease and improve patient survival. In the medical field, there are data that, for various reasons, are lost. There are also datasets that mix numerical and categorical values. Very few algorithms classify datasets with such characteristics. Therefore, this study proposes the modification of an existing algorithm for the classification of cancer. The said algorithm showed excellent results compared with classical classification algorithms. The AISAC-MMD (Mixed and Missing Data) is based on the AISAC and was modified to work with datasets with missing and mixed values. It showed significantly better performance than bio-inspired or classical classification algorithms. Statistical analysis established that the AISAC-MMD significantly outperformed the Nearest Neighbor, C4.5, Naïve Bayes, ALVOT, Naïve Associative Classifier, AIRS1, Immunos1, and CLONALG algorithms in conducting breast cancer classification.

## 1. Introduction

Cancer is a global problem that causes one in four deaths [1]. In men, the three most common cancers are lung, colon, and prostate, while in women, the most common cancers are breast and colorectal.

There are more than 27 different types of cancer [2], which is alarming as it is the second leading cause of death worldwide. The development of this disease is based on various criteria, such as gender, genetics, and race, among others [3]. Using non-invasive techniques allows medics and researchers to identify cancer early, allowing better treatment for patients, thereby saving lives.

For breast cancer, the pre-diagnosis process may vary according to the type and stage of cancer. However, some non-invasive studies are based on obtaining a digital image through a study (magnetic resonance, mammography, etc.) and then segmenting the region of interest (lesion). The characteristics of the lesion are obtained, and finally, the image is classified.

Several algorithms have been used for cancer classification. Due to the “No free lunch theorem” [4], there is no perfect classification algorithm; therefore, research on breast cancer classification continues to be an area of interest [5,6,7,8,9,10,11].

In this study, we use a metaheuristic based on the human immune system; this is an algorithm that imitates the behavior of fauna or a biological system to solve computational problems [12]. Due to their behavior, these algorithms are commonly used to solve non-deterministic problems since they are based on guiding a random solution in a defined search space [13,14].

It is important to emphasize that in medical datasets, mixed data are common; that is, data consisting of categorical and numerical values. Values may also be missing due to various factors. This is relevant given that most clinical data require pre-classification treatment.

In this study, we will work on the classification task, for which we propose a classification algorithm based on the human immune system. Currently, some classifiers work with mixed data. To the best of our knowledge, none of these algorithms is bio-inspired. However, bio-inspired models have been beneficial and widely used in medical diagnosis. For this reason, we propose a bio-inspired classification algorithm that can handle mixed and incomplete data.

This paper makes several contributions. We designed an Artificial Immune System for Associative Classification of Mixed and Missing Data (AISAC-MMD). This is a novel, immune-based classification algorithm that allows native dealing with multiclass, mixed, and incomplete data. This algorithm has low computational complexity.

The statistical analysis carried out established that the AISAC-MMD significantly outperformed the Nearest Neighbor, C4.5, Naïve Bayes, ALVOT, Naïve Associative Classifier, AIRS1, Immunos1, and CLONALG algorithms in classifying breast cancer.

The paper is structured as follows: Section 2 briefly addresses some of the previous works on computationally assisted breast cancer classification and pre-diagnosis. Section 3 explains the materials and methods used. Section 4 presents the results, detailing the newly proposed classification algorithm, while Section 5 discusses the numerical performance of the AISAC-MMD with respect to state-of-the-art classification algorithms. The paper ends with the conclusions and directions for future study.

## 2. Related Works

Over the last 5 years, research has been published on breast cancer pre-diagnosis using classification algorithms, such as the work of Amrane et al. [5], which tested KNN and Naïve Bayes algorithms applied to breast cancer classification for binary datasets. The results revealed that KNN yielded better accuracy than Naïve Bayes for breast cancer classification.

In 2019, Saritas and Yasar [6] analyzed classification algorithms (Artificial Neural Networks and Naïve Bayes) applied to the classification of breast cancer using biomarkers. The results showed excellent performance of these two algorithms, with Artificial Neural Networks obtaining the greatest accuracy. In the same year, Ting et al. [7] proposed Convolutional Neural Networks for breast cancer classification using medical images. The results revealed high classification accuracy. Their work was tested on a real dataset of 221 patients classified into three groups (malignant, benign, and healthy).

Numerous studies have examined the classification of breast cancer; however, this is not only cancer to be pre-diagnosis. For example, some papers, such as the recent work of Yuan et al. in 2019, used a classification method based on a magnetic resonance model to classify a dataset of patients with prostate cancer [8]. The model yielded good results in treating and classifying magnetic resonance images for prostate cancer.

In early 2020, Acharya et al. [9] proposed a combination of enhancing image pre-processing and deep learning algorithms to improve the classification of algorithms applied to breast cancer datasets. This modification showed better accuracy for the classification algorithms tested. A similar approach was proposed by Arif et al. (2020) [10], who reviewed deep learning approaches for classifying prostate cancer using magnetic resonance images. They concluded that new validations and clinical studies should be conducted to obtain better decision-making algorithms.

In 2020, Devarriya et al. [11] proposed two fitness functions for Genetic Programming. These were used for breast cancer classification, and showed good performance with imbalanced datasets. The first approach was based on learning about the minority class, while the second approach was based on according the same importance to both classes. Based on reviews conducted in our previous works, there are opportunities for improvement. This study proposes modifying a classification algorithm based on the human immune system, demonstrating promising results.

An interesting proposal based on bio-inspired algorithms is put forward by González-Patiño et al. [15], yielding promising results for breast cancer classification. Recently, deep learning has been analyzed, and has been reported as a useful tool for this task [16,17,18]. In addition, there has been an increase over the past year in the use of bio-inspired techniques for automatic breast cancer detection [19,20,21].

However, the above-mentioned proposals only deal with numeric and complete data. Therefore, these methods need to take the additional step of data pre-processing to impute (or even delete) missing records, and to change categorical values into numeric ones. Such procedures alter the nature of the data and can lead to poor performance. This study aims to overcome these drawbacks by designing a novel algorithm that is able to natively deal with mixed and missing data.

## 3. Materials and Methods

This section describes the datasets, performance measures, and algorithms that were compared. Nine algorithms were tested for the classification of ten datasets.

### 3.1. Datasets

In this study, we used ten datasets related to different types of cancer. It is important to note that the datasets contained missing and mixed values, which is quite common in medical datasets.

1. Breast Cancer Digital Repository (BCDR) [22]. This dataset is composed of data extracted from Portuguese women after being tested with biopsies to identify breast lesions. As stated in [22], “BCDR-F01 has a total of 362 segmentations from which 187 are from benign findings and the remainder 175 from malignant findings. In addition to the patient age and breast density, the data set includes a set of selected binary attributes for indicating abnormalities observed by radiologists, namely masses, microcalcifications, calcifications (other than microcalcifications), axillary adenopathies, architectural distortions, and stroma distortions. Thus, the clinical data for each instance of the BCDR-F01 data set include a total of eight attributes per instance: six binary attributes related to observed abnormalities, an ordinal attribute for breast density, and a numerical attribute that contains the patient age at the time of the study.”
2. Breast Cancer Wisconsin (Original) Data Set (BCWO) [23]. This dataset was provided by the UCI repository [24] and is available at http://archive.ics.uci.edu/ml/datasets/Breast+Cancer+Wisconsin+%28Original%29, accessed on 11 January 2021. It consists of patients treated by Dr. Wolberg, offering valuable information on clinical cases of breast cancer. BCWO contains 699 records of tissue samples, with each record characterized by the following attributes: Clump Thickness, Uniformity of Cell Size, Uniformity of Cell Shape, Marginal Adhesion, Single Epithelial Cell Size, Bare Nuclei, Bland Chromatin, Normal Nucleoli, and Mitoses. All attributes were manually measured on a scale of 1 to 10.
3. Breast Cancer SEER (BCSEER) [25]. The National Cancer Institute provides this dataset, which consists of real patients from 1973 to 2013 who underwent breast cancer-related studies. The institute provides the surveillance, epidemiology, and end results (SEER) database. The SEER database classifies cancer histology and topography information based on the third edition of the *International Classifications of Diseases for Oncology (ICD-O-3)*. In our study, we used the version of the dataset available on the Kaggle website (https://www.kaggle.com/code/jnegrini/breast-cancer-dataset, accessed on 11 January 2021).
4. Breast Cancer Wisconsin (Diagnostic) Data Set (BCWD) [26]. This binary dataset, provided by Dr. Wolberg in 1995, consists of data obtained from breast analysis and subsequently confirmed by biopsy. Features are computed from a digitized image of a fine needle aspirate (FNA) of a breast mass. They describe the characteristics of the cell nuclei present in the image. These features include radius (mean of distances from center to points on the perimeter), texture (standard deviation of gray-scale values), perimeter, area, smoothness (local variation in radius lengths), compactness (perimeter^2/area − 1.0), concavity (severity of concave portions of the contour), concave points (number of concave portions of the contour), symmetry, and fractal dimension (“coastline approximation” − 1). The dataset is available at http://archive.ics.uci.edu/ml/datasets/Breast+Cancer+Wisconsin+%28Diagnostic%29, accessed on 11 January 2021.
5. Breast Cancer Wisconsin (Prognostic) Data Set (BCWP) [27]. This dataset was provided by Dr. Wolberg and contained data on breast cancer patients with invasive breast cancer. This dataset was donated in the same year as the BCWD. Each record represents follow-up data on one breast cancer case. These are consecutive patients seen by Dr. Wolberg since 1984 and include only those cases exhibiting invasive breast cancer and no evidence of distant metastases at the time of diagnosis. The dataset has 32 predictive attributes, with the first 30 computed from a digitized image of a fine needle aspirate (FNA) of a breast mass. They describe the characteristics of the cell nuclei present in the image. The other two attributes are recurrence time (in case of recurrence) and disease-free time (in case of non-recurrence). This dataset is available at http://archive.ics.uci.edu/ml/datasets/Breast+Cancer+Wisconsin+%28Prognostic%29, accessed on 11 January 2021.
6. Lung Cancer Data Set (LCDS) [18]. This dataset was chosen as it contains information on patients who had surgeries. The dataset, which was donated in 1999, focuses on the survival of these patients after surgery. It is an interesting dataset due to the scarcity of the data (only 32 subjects) and the large amount of predictive features (55). It is available at http://archive.ics.uci.edu/ml/datasets/Lung+Cancer, accessed on 11 January 2021.
7. Mammographic Mass Data Set (MMDS) [28]. Donated in 2007, this dataset contains patterns of mammography studies carried out on 961 German patients. It contains a BI-RADS assessment, the patient’s age, and three BI-RADS attributes. It also contains the ground truth (severity field) for 516 benign and 445 malignant masses identified on full-field digital mammograms, collected at the Institute of Radiology of the University Erlangen-Nuremberg between 2003 and 2006. Each instance has an associated BI-RADS assessment ranging from 1 (definitely benign) to 5 (highly suggestive of malignancy) assigned in a double-review process by physicians. The dataset is available at http://archive.ics.uci.edu/ml/datasets/Mammographic+Mass, accessed on 11 January 2021.
8. Breast Cancer Data Set (BCDS) [29]. This dataset, which contains data on patients with recurrent breast cancer, was provided by Milan Soklic and Matjaz Zwitter at the Institute of Oncology in Yugoslavia. The dataset contains eight attributes: age, menopause, premenopausal, tumor size, inv-nodes, node-caps (yes, no), degree of malignancy (1, 2, 3), breast (left, right), breast quad (left-up, left-low, right-up, right-low, central), and irradiation (yes, no). The dataset is available at http://archive.ics.uci.edu/ml/datasets/Breast+Cancer, accessed on 11 January 2021.
9. Haberman’s Survival Data Set (HSDS) [30]. This dataset was donated in 1999 to the Machine Learning repository of the University of California [18]. It contains data on the survival of patients with breast cancer who had surgical removal of lesions. It has only four predictive features: age of patient at the time of operation (numerical), patient’s year of operation, and number of positive axillary nodes detected. The decision attribute is survival status (1 if the patient survived 5 years or longer or 2 if the patient died within 5 years). The dataset is available at http://archive.ics.uci.edu/ml/datasets/Haberman%27s+Survival, accessed on 11 January 2021.
10. Thoracic Surgery Data Set (TSDS) [31]. The data was collected retrospectively at the Wrocław Thoracic Surgery Centre for patients who underwent major lung resections for primary lung cancer in the years 2007–2011. The Centre is associated with the Department of Thoracic Surgery of the Medical University of Wrocław and the Lower-Silesian Centre for Pulmonary Diseases, Poland. The research database constitutes a part of the National Lung Cancer Registry, administered by the Institute of Tuberculosis and Pulmonary Diseases in Warsaw, Poland. The goal of the dataset is to predict whether the patient will or will not survive surgery. The dataset has 16 predictive attributes: forced vital capacity; volume that has been exhaled at the end of the first second of forced expiration; performance status (Zubrod scale); pain before surgery; hemoptysis before surgery; dyspnea before surgery; cough before surgery; weakness before surgery; size of the original tumor, from OC11 (smallest) to OC14 (largest); type 2 DM—diabetes mellitus; MI up to 6 months; peripheral arterial diseases; smoking; asthma; and age at surgery. The dataset can be found at http://archive.ics.uci.edu/ml/datasets/Thoracic+Surgery+Data, accessed on 11 January 2021.

Table 1 summarizes the most relevant characteristics of each dataset.

We considered the existence of missing values, the number of instances and attributes, and the imbalance ratio (IR). A dataset is considered imbalanced if the IR measure exceeds 1.5 [32]. All datasets had two classes except for the LCDS dataset, which had three.

### 3.2. Algorithms

Eight algorithms were selected. The first five algorithms were chosen since they work with mixed and missing data, which is one of the main contributions of the proposed model in this study. The following three algorithms were based on the same principle as the proposed model; that is, they on an Artificial Immune System. This is why they were selected for comparison against other algorithms of the same type.

1. K-Nearest Neighbors (NN) was proposed by Cover and Hart in 1967 [33]. This algorithm is based on assigning a class according to the k nearest pattern. If the pattern belongs to different classes, a majority voting process will be carried out to obtain a single class.
2. C4.5 [34] was developed as a modification of ID3 [35]. It is a decision tree for making decisions based on relevant information provided by each attribute.
3. Naïve Bayes [36] is a classifier based on probability and the independence of each attribute. It is derived from Bayes’ theorem.
4. ALVOT is a general purpose classification model that uses different views of information based on a Support Set System [37]. This model uses a voting schema based on aggregation procedures. The model has a high computational cost when using all typical testors, but it can obtain good results with mixed and incomplete data.
5. NAC was proposed in 2017 by Villuendas-Rey et al. [38] as a learning model for classifying mixed and incomplete data. It is based on a similarity operator named MIDSO, and is a particular case of both the ALVOT and NN classifiers. It has low computational complexity and yields good results when applied to financial data.
6. AIRS1 is a classification algorithm based on the Artificial Immune System, The algorithm was proposed in 2001 [39], based on the principle of clonal selection and affinity maturation.
7. Immunos1 is another algorithm that reduces information in one training iteration. It was proposed in 2005 [40].
8. CLONALG is an algorithm based on the principle of clonal selection for classification. Each prototype improves the recognition of patterns in each iteration due to the affinity function. This algorithm was proposed in 2002 [41].

It should be noted that these last three algorithms do not operate with missing or mixed values, which is why an imputation was necessary. Table 2 shows the parameters of the compared algorithms; we used the default parameters, as proposed in the original implementations.

### 3.3. Performance Measure

Due to data imbalances, we used the Balanced Accuracy measure, also known as macro average accuracy [42]. Balanced Accuracy is based on calculating each class’s accuracy and subsequently averaging that accuracy.

**Figure 1:**
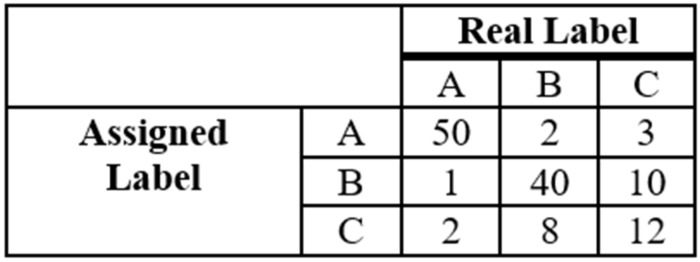
Example of a Confusion matrix for three classes.

This measure can be easily calculated if we use the Confusion matrix, which presents correctly classified patterns for each class. Figure 1 shows an example of a Confusion matrix for three classes.

The general formula for Balance Accuracy is presented in Equation (1), where $S_{i}$ is the Recall of the class i, and k is the number of classes.

(1) $$\mathrm{Balanced}\;\mathrm{Accuracy}\;=\left(\sum_{i=1}^{k}S_{i}\right)/k$$

## 4. Results

Our proposal is based on the recently introduced Artificial Immune System for Associative Classification (AISAC) [15]. Our aim was to address AISAC’s main drawback of not working with missing or mixed data (MMD), given that several medical datasets have these characteristics. Based on the AISAC, we proposed modifications that yielded better performance. Thus, we offered a solution to problems associated with the AISAC through a novel algorithm named the Artificial Immune System for Associative Classification in Mixed and Missing Data (AISAC-MMD).

**Figure 2:**
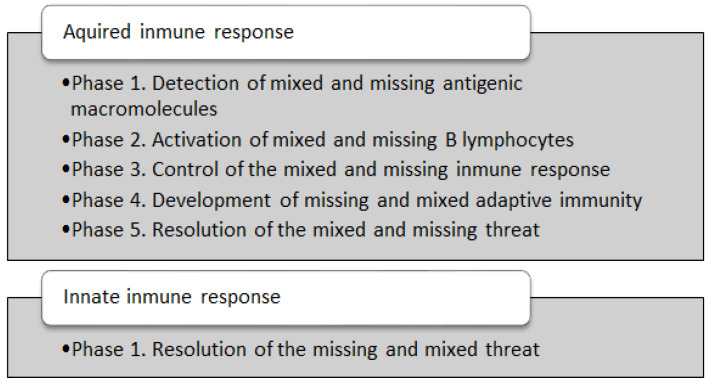
Immune response in the AISAC-MMD model.

The proposed algorithm incorporates several modifications of MMD, as shown in Figure 2.

To explain the variants and modifications introduced in the proposed AISAC-MMD, we use the pseudocode presented in Figure 2 to better explain the changes in each phase.

**Figure 3:**
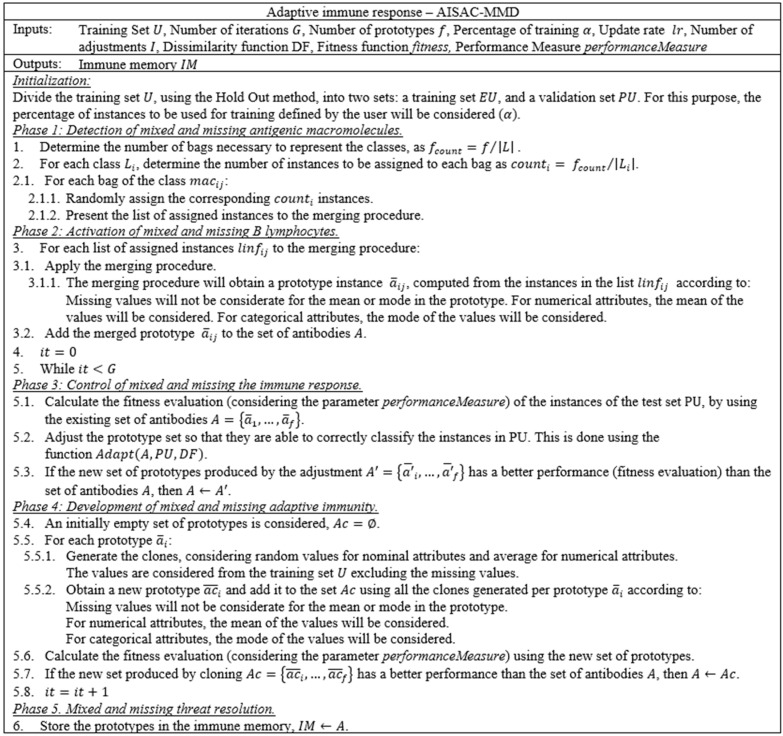
Pseudocode of the adaptive immune response in the AISAC-MMD model.

In Figure 3, we present the modification of the Adaptive Immune Response, which uses missing and mixed data. With regard to data structures, we stored the training set as a list of instances, and consider that each instance has a decision class. We required a dissimilarity function to compare two instances (user-defined), a fitness function to assess the quality of the created prototype set, and the associated performance measure (used-defined).

We start by dividing the training set by Hold-Out. Then, we will create several clusters (bags) to initially structure the data (Phase 1). In Phase 2, we merge the instances in the bags, thereby obtaining the initial prototype set to represent the data. After that, the algorithm undergoes an iterative process (Phases 3 and 4). Phase 3 “moves” the instances in such a way that the performance measure is optimized. After that, to avoid overfitting, Phase 4 creates clones and obtains a new set of prototypes. At the end of the iterative process, the algorithm stores the final prototype set in memory.

**Figure 4:**
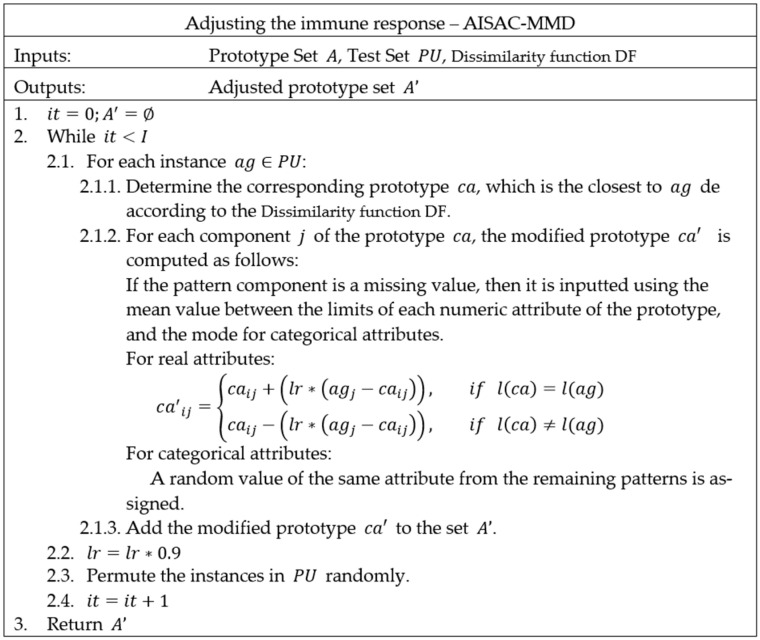
Pseudocode of the adjustment in the adaptive immune response in the AISAC-MMD model.

For the distance calculation, we set a parameter for the Dissimilarity function. In our experiments, we use the HEOM dissimilarity. Similarly, we modified the Adjusting function (Adapt), as presented in Figure 4, in which we changed the dissimilarity function.

In cases of patterns with missing values, which are selected as the closest elements for a specific pattern in any part of the algorithm, for the computation process of the prototype, the missing values are substituted by the mean value for numeric attributes or by the mode for categorical attributes. This allows us to update the prototypes without modifying the original patterns.

This is the first bio-inspired classifier that works with mixed and missing information without transforming the data. In other words, the AISAC-MMD maintains the missing and mixed values without imputing the attributes and including artificial values. It will be beneficial in the medical field since most datasets have these characteristics.

The following section discusses the comparison between the proposed AISAC-MMD and existing classifiers.

## 5. Discussion

We used the 10 datasets described in Section 3.1 to assess the performance of the AISAC-MMD. The experiments were conducted on a desktop computer with a 64 bit Windows 10 Enterprise operating system, an Intel i5-6500 processor at 3.20 GH, and 16 GB of RAM. As this was a work computer, all experiments were carried out under low priority.

We compared the datasets with the nine classification algorithms for breast cancer-related prediction. First, we compared the AISAC-MMD against classical classification algorithms that work with mixed data and missing values. The results are presented in Table 3. We used a Balanced Accuracy measure (Equation (1)) due to the high degree of imbalance present in the datasets (Table 1). In this way, we managed to avoid bias toward the majority classes. The AISAC-MMD obtained the best performance for seven out of ten datasets, compared with other algorithms that work with missing and mixed values. The best performance for each dataset is highlighted in bold.

The second comparison was performed on algorithms based on artificial immune systems (Table 4). Again, the best results for each dataset are indicated in bold.

Regarding algorithms based on the same principle, the AISAC-MMD obtained the best performance for nine datasets. With these results, we proceeded to perform a statistical test.

We conducted the Wilcoxon test, which identifies the presence or absence of differences in performance between various algorithms. This test is based on selecting an algorithm and comparing it with another. In this case, we compared the new AISAC-MMD model with other algorithms.

The statistical test (Wilcoxon test) to compare the algorithms in the same datasets is presented in the next section. This test is widely used to identify differences in performances comparing several algorithms [43].

The Wilcoxon signed-rank was used in this study. The comparison is presented in Table 5, considering α=0.05, which means values lower than that represent the rejection of the null hypothesis H0. Hypothesis H0 states that there are no differences in the performance of the compared algorithms. We set a confidence level of 95%. We first performed the test to compare the AISAC-MMD against the classical algorithms that work with missing and mixed data (Table 5).

Concerning the literature algorithms, the null hypothesis H0 was rejected in all algorithms. Therefore, the AISAC-MMD outperformed these algorithms. These algorithms are based on the same principle as the Artificial Immune System, and the AISAC-MMD performed better, as demonstrated by the statistical test.

In summary, the AISAC-MMD outperformed all eight classification algorithms. Comparing the new modification with its previous version, the AISAC-MMD performed well, in addition to working with mixed data and missing values.

## 6. Conclusions

In this study, we introduced the first bio-inspired classification algorithm that is able to natively deal with missing and mixed data. The advantages of this algorithm are:

Its ability to handle missing and mixed data without any pre-processing; this is useful since most datasets present missing values and mixed attributes.

1. Its creation of a reduced prototype set; this decreases storage complexity, making it suitable for hardware implementation in devices associated with other medical devices, such as mammographs, etc.
2. Its ease of use and good performance, which allows doctors to make decisions when there is high demand in the analysis of mammographic studies.
3. The main limitation of the proposal is that, as with most metaheuristics, it has several parameters. This helps to improve the algorithm’s performance by varying the values of the parameters.

In this study, no parameter adjustment was performed nor were different configurations tested. This aspect can be examined in future research to improve the performance of the algorithm. Finally, the use of other strategies can be examined to further explore this research area.

## Figures and Tables

**Table 1:** Summary of the characteristics of the datasets.

Dataset	Attributes	Instances	Imbalance Ratio	Missing Values
BCDR	38	362	1.06	Yes
BCWO	9	699	1.90	Yes
BCSEER	5	1405	5.41	No
BCWD	30	569	1.60	No
BCWP	33	198	3.21	Yes
LCDS	56	32	1.44	Yes
MMDS	5	961	1.15	Yes
BCDS	9	286	2.36	Yes
HSDS	3	306	2.77	No
TSDS	14	470	5.71	No

**Table 2:** Parameters of the algorithms.

Algorithm	Parameters
NN	K: 1; Dissimilarity: HEOM
C4.5	BinarySplits: False; collapseTree: True; confidenceFactor: 0.25; minNumObj: 2; numFolds: 3; unpruned: False; useLaplace: False; useMDLcorrection: True;
Naïve Bayes	-
ALVOT	Dissimilarity: HEOM, Support Set System: All attributes
NAC	Dissimilarity: HEOM
AIRS1	seed = 1; affinityThresholdScalar = 0.2; mutationRate = 0.1; totalResources = 150; stimulationValue = 0.9; clonalRate = 10; hypermutationRate = 2.0; numInstancesAffinityThreshold = −1; arbInitialPoolSize = 1; memInitialPoolSize = 1; knn = 3;
Immunos1	-
CLONALG	clonalFactor = 0.1; antibodyPoolSize = 30; selectionPoolSize = 20; totalReplacement = 0; numGenerations = 10; seed = 1; remainderPoolRatio = 0.1

**Table 3:** Balanced accuracy results for classifiers dealing with mixed and incomplete data.

Dataset	ALVOT	C4.5	NAC	Naïve Bayes	NN	AISAC-MMD
BCDR	0.770	0.749	0.678	0.727	0.729	**0.784**
BCWO	0.941	0.951	**0.975**	0.960	0.953	0.969
BCSEER	0.834	**1.000**	0.908	0.972	0.984	**1.000**
BCWD	0.934	0.931	0.894	0.930	0.960	**0.965**
BCWP	0.563	0.727	0.699	0.667	0.707	**0.767**
LCDS	0.542	0.469	0.450	0.594	0.531	**0.688**
MMDS	0.789	**0.823**	0.806	0.778	0.752	0.797
BCDS	0.728	**0.741**	0.731	0.727	0.682	0.731
HSDS	0.748	0.703	0.733	0.748	0.660	**0.765**
TSDS	0.728	**0.845**	0.774	0.745	0.760	**0.845**

**Table 4:** Balanced accuracy results for classifiers based on artificial immune systems.

Dataset	AIRS1	CLONALG	Immunos1	AISAC-MMD
BCDR	0.732	0.577	0.561	**0.784**
BCWO	0.967	0.941	0.847	**0.969**
BCSEER	0.945	0.965	0.954	**1.000**
BCWD	0.938	0.889	0.905	**0.965**
BCWP	0.641	0.742	0.566	**0.767**
LCDS	0.531	0.469	0.563	**0.688**
MMDS	0.634	0.700	0.743	**0.797**
BCDS	0.675	0.671	**0.734**	0.731
HSDS	0.637	0.732	0.568	**0.765**
TSDS	0.774	0.745	0.760	**0.845**

**Table 5:** Results of the Wilcoxon test.

AISAC-MMD vs.	R+	R−	*p*-Value	Decision
NN	55	0	0.004317	Reject H0
C4.5	49	6	0.024932	Reject H0
Naïve Bayes	55	0	0.004317	Reject H0
ALVOT	39	6	0.044011	Reject H0
NAC	55	0	0.004317	Reject H0
AIRS1	55	0	0.004317	Reject H0
Immunos1	54	1	0.005922	Reject H0
CLONALG	55	0	0.004317	Reject H0

## Data Availability

All datasets are publicly available from the Machine Learning Repository of the University of California at Irvine [18] (https://archive.ics.uci.edu/ml/datasets.php, accessed on 11 January 2021) except the ones in [22], which are available upon request.
